# Who would own the HeLa cell line if the Henrietta Lacks case happened in present-day South Africa?

**DOI:** 10.1093/jlb/lsad011

**Published:** 2023-05-16

**Authors:** Donrich W Thaldar

**Affiliations:** School of Law, University of KwaZulu-Natal, Durban, South Africa; Petrie-Flom Center for Health Law Policy, Biotechnology, and Bioethics at Harvard Law School, Cambridge, MA, USA

**Keywords:** HeLa, ownership, South Africa, law, informed consent

## Abstract

The HeLa cell line was created in 1951 without consent from Henrietta Lacks, the person whose tissue sample was used. In 2021, the descendants of Henrietta Lacks sued a well-known biotechnology company for the profits it made from the HeLa cell line. In this article, ownership of the cell lines is investigated from a South African legal perspective by considering three possible contemporary scenarios bearing points of similarity to the Henrietta Lacks case. In the first scenario, informed consent is obtained to use a tissue sample for research and to commercialize the products of such research; in the second scenario, informed consent is materially deficient because of an honest mistake on the part of the research institution; and in the third scenario, informed consent is materially deficient due to willful disregard of the law on the part of the research institution. In the first two scenarios, ownership of the cell line created from the tissue sample would vest in the research institution, and the research participant would not have any legal action for financial compensation. However, in the third scenario, ownership of the cell line would vest in the research participant, who would be able to claim all profits made from trading the cell line. Whether the research institution acted in good faith is therefore a crucial determinant of the legal outcome.

## I. INTRODUCTION

The case of Henrietta Lacks is well known.^1^ In 1951, she was diagnosed with cancer and was undergoing treatment at Johns Hopkins Hospital in the United States. A tissue sample retrieved during a biopsy was sent to the research laboratory of cell biologist Dr George Otto Gey, who analyzed retrieved cells from patients. From this sample, Gey’s laboratory assistant isolated the cancer cells. These cells proved to be different from all previous cells studied in Gey’s laboratory. By applying a technique developed by Gey, these cells could grow and divide indefinitely in culture medium. Using Lacks’ cancer cells, Gey developed the world’s first immortal human cell line, which he called the ‘HeLa’ cell line after Henrietta Lacks. Gey distributed the HeLa cell line widely and free of charge. Over the past 70 years, the HeLa cell line was used in countless health-related innovations, ranging from the polio vaccine in the 1950s to studying the human genome and more recently the COVID-19 vaccines. Almost 11,000 patents are involved in the HeLa cell line. The problem, however, is that Henrietta Lacks’ consent was never obtained to use the retrieved cells for research purposes. Although the actions that took place were accepted practice 70 years ago, society’s values have since changed. As such, the HeLa cell line has become controversial. The history of the HeLa cell line raises several ethical and legal issues. One central issue is the *ownership* of the HeLa cell line. This issue is sure to be canvassed in the lawsuit filed at the end of 2021 by the estate of Henrietta Lacks against Thermo Fisher Scientific; it is likely that all profits made by the biotech company from commercializing the HeLa cell line will be claimed.^2^

In this essay, I investigate the ownership of the cell lines through the lens of South African law. I pose the hypothetical question: if a case similar to that of Henrietta Lacks occurred in present-day South Africa, who would own the HeLa cell line? As a secondary question, I consider possible private law remedies that a present-day South African Henrietta Lacks may have at her disposal. Given the differences between present-day South Africa and 1950s America, the exact facts of the Henrietta Lacks case cannot simply be superimposed on present-day South Africa. This is illustrated by the following observations: contrary to the Henrietta Lacks case, consent for using her human biological material (HBM) in research is a legal requirement in present-day South Africa. Also, contrary to Gey who presumably believed—correctly so—that he did not break the law in any way, it would not be plausible for present-day South African researchers to believe that no consent is required. And, as I show in my analysis below, a researcher’s honesty regarding the consent requirement is a crucial consideration in the law. Accordingly, I present three possible scenarios regarding research participant consent—or the lack thereof—in current South African law. These three scenarios are: (i) legal compliance, (ii) legal non-compliance because of an honest mistake, and (iii) willful legal non-compliance by research institutions with informed consent legal requirements. The reader can compare the legal positions of these three scenarios.

Before I can commence with the analysis of the three scenarios, I first need to answer the foundational—and sometimes contested^3^—question of whether HBM in the research context is susceptible of ownership in South African law.

## II. CAN HBM BE OWNED?

Two factors contribute to the lack of consensus about the legal status of HBM in the research context. First, the South African courts have not yet had the opportunity to apply common law principles to the issue of ownership of HBM in the research context.^4^ Second, South African statutory law in the area of health research is fragmented, sometimes contradictory, and mostly not explicit on the issue. In a recent article,^5^ Thaldar and Shozi present the first comprehensive analysis of South African law on the topic and conclude that HBM in the research context is indeed susceptible of ownership. Their analysis is summarized in the following two paragraphs.

### Common Law

South Africa’s common law—Roman-Dutch law—is a flexible system that develops with the times. The abstract principle in Roman-Dutch law is that anything that is useful and valuable, not part of something else or of a human body, and that is capable of human control, qualifies as a legal object that is susceptible of ownership. Accordingly, although a tissue sample might not have been susceptible of ownership in Roman times (because the Romans did not have much use for it), a tissue sample that is currently used for research is indeed susceptible of ownership because it is (a) useful and valuable in research, (b) removed and hence separated from a human body, and (c) capable of human control. This interpretation is supported by judgments in jurisdictions that share the same principles as found in Roman-Dutch law.^6^

### Statute Law

South Africa’s statute law necessarily implies ownership of HBM in the research context. The National Health Act^7^ (NHA) and its regulations^8^ consistently use the term ‘donation’ to denote a situation where certain kinds of HBM (tissue, gametes, blood, or blood products) or HBM generally are provided by a research participant to a research institution for the purposes of research. ‘Donation’ is a legal technical term that entails that *ownership* of the donated object is transferred from the donor to the donee.^9^ This means that the research participant is the *original owner* of HBM originating from his or her body, and that the research participant *transfers ownership* of the HBM to the research institution to which the HBM is donated. Importantly, the NHA provides (in section 55) that it is unlawful to provide human tissue for research in any way other than the way provided for in the NHA. Accordingly, human tissue samples may not be provided by a research participant for research in any way other than *donation—*i.e., transfer of ownership is peremptory.

In the next two paragraphs, I address two common arguments against the conclusions reached above. Also, since HBM contain genetic information, I also consider whether South Africa’s data protection legislation has any effect on these conclusions.

### But if one May Not Trade in HBM, how Can it Be Owned?

The answer to this question has two parts. First, the fact that there are certain restrictions on what owners may lawfully do with their property does not mean that there is no ownership. For example, the fact that one may not trade in cannabis does not mean that one cannot own cannabis.^10^ The same applies to HBM. Second, the claim that one may not trade in HBM is not entirely true. The NHA’s trade restrictions only apply to the quartet of tissue, blood, blood products, or gametes—not to HBM generally.^11^ Nothing in the definition of ‘tissue’ in the NHA suggests that it includes objects of a new kind that can be derived from tissue, such as cell lines. It is important to note that legal rules that are applicable to one kind of object are not necessarily applicable to the kinds of objects that can be derived from the former kind. For example, a legal rule that televisions must be licensed would be applicable to televisions, but not to a component such as the metal housing or to recycled metal from the television. Similarly, a cell line is a different kind of legal object from tissue, and hence is not included in the trade ban on tissue.

### Can Research Participants Retain Ownership?

In 2018, the South African Minister of Health promulgated a material transfer agreement (MTA) to be used whenever biospecimens are transferred for purposes of research or clinical trials within or out of South Africa (SA MTA).^12^ In contrast with the statutes discussed above, the SA MTA provides that ‘the donor remains the owner’ of the HBM. However, this provision has been highlighted as problematic.^13^ First, the provision does not make sense, as donation would not be donation without the transfer of ownership. Without the transfer of ownership, the nature of the transaction would be something else, such as lending. Second, the Minister of Health can only make regulations within the framework provided by the NHA; the Minister of Health cannot change the provisions of the NHA. The NHA makes the transfer of ownership peremptory, and any provision to the contrary in the SA MTA is therefore null and void. It should also be noted that the SA MTA itself states that it is only a ‘framework’. As such, users of the SA MTA are free to change any of the substantive provisions of the SA MTA as they wish—as long as the main headings are retained in some form.^14^ A revised version of the SA MTA that retained the original framework but eliminated this and other problematic issues in the original SA MTA was developed by a group of law academics and is freely available online.^15^

### What about POPIA?

South Africa’s data protection legislation, the Protection of Personal Information Act 4 of 2013 (POPIA)^16^ entered into force on 1 July 2021. POPIA provides for comprehensive informational privacy rights for persons to whom personal information relate—i.e., data subjects. Given that HBM contains genetic information about a person, and genetic information qualifies as personal information, does POPIA not apply to such HBM? The answer is ‘no’.^17^ The reason is that POPIA only applies to personal information that is ‘entered in a record by or for a responsible party’ [section 3(1)]. Genetic information contained in HBM naturally occurs in such HBM, rather than being ‘entered in’ such HBM ‘by or for a responsible party’. Accordingly, HBM and the genetic information that it contains fall outside the scope of POPIA.

Now that it has been established that HBM in the research context is susceptible of ownership, that the transfer of ownership by the research participant to the research institution is peremptory, and that HBM and the information contained therein are not regulated by POPIA, I consider the three scenarios.

## III. PRESENT-DAY SCENARIOS

### Scenario 1—Legal Compliance

The NHA provides (section 55 read with section 71) that tissue samples may only be taken with the written informed consent of the research participant. In the first scenario, it is assumed that all involved act lawfully, and the hypothetical present-day Henrietta Lacks gives informed consent in writing for the removal of a tissue sample. Her informed consent is sufficiently comprehensive to cover research using the sample, and the commercialization of products from such research. In this scenario, it should be clear that ownership of the tissue sample is automatically, through the operation of the NHA, transferred to the research institution. If the research institution isolates the individual cells from the tissue sample and creates a cell line from the tissue sample, the default position would be that the research institution is the owner of the cell line. Since cell lines fall outside the NHA’s trade ban, the research institution would be free to commercialize the cell line.

### Scenario 2—Legal Non-Compliance Because of an Honest Mistake

In the second scenario, the informed consent provided by the hypothetical present-day Henrietta Lacks is defective in a material way because of an honest mistake on the part of the research institution. For example, if the research institution mistakenly makes an incorrect representation to present-day Henrietta Lacks regarding a material aspect of the transaction, there would not be true consent, and the donation would be unlawful and hence void. An example of this would be if the research institution represented to present-day Henrietta Lacks that she would retain ownership of her donated tissue sample, while in fact (because of the restriction imposed by the NHA) it is a legal impossibility. Ironically, the result would be that this false representation would become true: since the donation is void, present-day Henrietta Lacks would remain the owner of the tissue sample. Assuming that the research institution makes the incorrect representation because it sincerely believes it to be legally possible, the research institution would become the *bona fide* possessor of the tissue sample. This is important, as the analysis below will show.

The next part of the analysis is to consider the creation of the cell line from the tissue sample from a property law perspective. Importantly, a cell line is a *new kind of object* that is created from the tissue sample. South African law provides a solution to this situation with the Roman law concept *specificatio*. This entails that where a person who is in good faith possession of an object but not the owner thereof, makes an object of a new kind out of the original object, and without an agreement between the maker and the owner, the maker acquires ownership in the new object.^18^ Classic examples are wine from another’s grapes, a statue from another’s gold, and clothes from another’s wool. Accordingly, in this scenario too, the research institution would become the owner of the cell line.

Would the present-day Henrietta Lacks have any remedy to financially compensate her for the loss of ownership? In South Africa’s common law, Roman-Dutch law, a person who lost ownership due to the operation of *specificatio*, would in principle be able to rely on an unjustified enrichment action to be compensated. For an unjustified enrichment action to be successful, the present-day Henrietta Lacks would need to prove, *inter alia*, that she was impoverished^19^ by the operation of *specificatio*. However, since the NHA places tissue outside the realm of trade, present-day Henrietta Lacks’ tissue sample had no patrimonial value, and its loss therefore had zero effect on her assets. As such, the present scenario would likely not be actionable by a present-day Henrietta Lacks.

### Scenario 3—Willful Legal Non-compliance

In the last scenario, the informed consent provided by the hypothetical present-day Henrietta Lacks is materially defective because the research institution acts in willful defiance of the law. This would be the case if, rather than *mistakenly* making an incorrect representation to the present-day Henrietta Lacks, the research institution makes the same representation knowing it to be false. Accordingly, although the research institution would be in *possession* of the tissue sample, the donation agreement would be unlawful and hence void, and *ownership* of the sample would not transfer to the research institution. *Specificatio* would not be applicable (as the research institution is not acting in good faith) and the present-day Henrietta Lacks would automatically be the owner of the cell line derived from her tissue sample.

In this scenario, the present-day Henrietta Lacks has two common law remedies. First, she can claim possession of the cell line from anyone who is in possession of it. (The legal description of the action would be *rei vindicatio*.) Second, she can claim all profits from anyone who sold the cell line, using a general enrichment action. (The legal description would be *condictio sine causa specialis*.) Why would an enrichment action succeed in this scenario, but not in the previous scenario? The reason is that in this scenario the present-day Henrietta Lacks is the owner of a *tradeable* legal object(s), namely the cell line. It follows that the profits made by others from her cell line ought to have been *her* profits, and the fact that she did not receive such profits means that she was impoverished. Accordingly, those who sell her cell line for profit enrich themselves at her expense, and she is entitled to claim such profits from them.

## IV. CONCLUSION

To encapsulate: where a research institution uses a tissue sample from a research participant to create a cell line, the research institution will be the owner of the cell line and the research participant will have no claim to any income generated by such cell line—provided that the research institution complied with the statutory informed consent requirements, or where the informed consent was deficient, it was an honest mistake on the part of the research institution. However, where the informed consent was deficient due to willful disregard of the law by the research institution, the research participant would be the owner of the cell line and would be entitled to all profits generated by the commercialization of the cell line.

It should be noted that classical Roman law was developed around the value of good faith, rather than based on strict compliance with rules. Via Roman-Dutch law, this more flexible approach is also part of South African law. Accordingly, strict compliance with the informed consent requirement of the NHA is not the only factor in South African law that determines ownership of the eventual cell line. The other factor is the honesty of the researchers involved—whether they acted in good faith.

I conclude with an observation regarding the US lawsuit brought by Henrietta Lacks’ estate against Thermo Fisher Scientific. At the time of writing, Thermo Fisher Scientific filed a motion to dismiss arguing that the case was brought outside the timeframe allowed by Maryland’s statute of limitations.^20^ If this case proceeds to court—and if it is not subsequently settled out of court—the court’s judgment would be an insightful addition to other leading US authorities on the rights of research participants vis-à-vis research institutions. It should be noted, however, that although South African courts may have regard for foreign judgments, such judgments are of course not binding authority in South Africa, and would only be relevant to the extent that the legal principles informing the judgment are applicable in South African law.^21^ Yet, what is true of both US and South African law—as both are largely uncodified systems of law—is that finding the legal position often requires an integration of various sources of the law. This includes the relevant jurisdiction’s statute law and common law, which may have its roots in centuries or millennia past. As illustrated by the analysis of the three scenarios above, problems brought about by modern science may have ancient solutions.

## Footnotes

1 Faroque A. Khan, *The Immortal Life of Henrietta Lacks*, 43 J. IMA. 93 (2011).

2 Ryan W. Miller, *Henrietta Lacks’ Cells Led to Medical Breakthroughs despite Her Lack of Consent. Now Her Family Is Suing a Pharmaceutical Company.*, USA Today (Oct. 4, 2021), https://www.usatoday.com/story/news/nation/2021/10/04/henrietta-lacks-family-sues-thermo-fisher-over-hela-cells-products/5987038001/; Catherine Offord, *Henrietta Lacks Estate Sues Thermo Fisher over HeLa Cell Line*, *The Scientist* (Oct. 4, 2021), https://www.the-scientist.com/news-opinion/henrietta-lacks-estate-sues-thermo-fisher-over-hela-69279 (Accessed on 3 April 2023).

3 Academy of Science of South Africa & Department of Science and Technology, *Human Genetics and Genomics in South Africa: Ethical, Legal and Social Implications* (2018), https://research.assaf.org.za/bitstream/handle/20.500.11911/106/2018_assaf_ethical_genetics_genomics_consensus.pdf?sequence=1&isAllowed=y; Safia Mahomed et al., *The Legal Position on the Classification of Human Tissue in South Africa: Can Tissues Be Owned?*, 6 S. Afr. J. Bioethics & L., 16 (2013); Donrich W. Jordaan, *Social Justice and Research Using Human Biological Material: A Response to Mahomed, Nöthling-Slabbert and Pepper*, 106 S. Afr. Med. J. 678 (2016); S. Mahomed et al., *Ownership and Human Tissue – the Legal Conundrum: A Response to Jordaan’s Critique*, 107 S. Afr. Med. J. 196 (2017); Donrich W. Jordaan, *An Imaginary Legal Conundrum: A Reply to the Response by Mahomed, Nöthling-Slabbert and Pepper*, 107 S. Afr. Med. J. 199 (2017); M.S. Pepper, *Exporting DNA – Striking a Balance between Preventing Exploitation and Promoting Innovation*, 107 S. Afr. Med. J. 106 (2017) .

4 Magda Slabbert, *This Is My Kidney, I Can Do What I Want with It — Property Rights and Ownership of Human Organs*, 30 Obiter 499 (2021).

5 Donrich W. Thaldar & Bonginkosi Shozi, *The Legal Status of Human Biological Material Used for Research*, 138 S. Afr. L. J. 881 (2021).

6
*R v. Kelly & Lindsay* [1999] QB 621 (Eng. & Wales); *Roche v. Douglas* [2000] WASC 146, (2000) 22 WAR 331 (Austl.); *CC v. AW* 2005 ABQB 290 (Can.); *Yearworth v. North Bristol NHS Trust* [2009] EWCA Civ. 37, [2010] QB 1 (Eng.).

7 National Health Act 61 of 2003 (S. Afr.).

8 Regulations Relating to the Use of Human Biological Material, GN R.177 of GG 35099 (2 Mar 2012) (S. Afr.); Regulations Regarding the General Control of Human Bodies, Tissue, Blood, Blood Products and Gametes, GN R.180 of GG 35099 (2 March 2012) (S. Afr.); Regulations Relating to Research with Human Participants, GN R.719 of GG 38000 (19 Sep 2014) (S. Afr.).

9 L.T.C. Harms, *DONATIONS* in The Law of South Africa (W.A. Joubert ed., 3 ed. 2017).

10
*Minister of Justice and Constitutional Development v. Prince* 2018 (6) SA 393 (CC) (S. Afr.).

11 Thaldar & Shozi, supra n. 5.

12 Material Transfer Agreement for Human Biological Materials, GN 719 of GG 41781 (20 July 2018) (S. Afr.).

13 Donrich W. Thaldar et al., *South Africa’s New Standard Material Transfer Agreement: Proposals for Improvement and Pointers for Implementation*, BMC Med. Ethics, Sept. 2020, 21; Donrich Thaldar, *One Material Transfer Agreement to Rule Them All? A Call for Revising South Africa’s New Standard Material Transfer Agreement*, Hum. & Soc. Sci. Comm., Sept. 2020, 7.

14 Thaldar & Shozi, supra n. 5.

15 Anne Pope et al., *South African Material Transfer Agreement 1.1*, (2020) https://researchspace.ukzn.ac.za/handle/10413/19095 (Accessed on 3 April 2023).

16 Protection of Personal Information Act 4 of 2013 (S. Afr.).

17 Donrich W. Thaldar & Beverley A. Townsend, *Exempting Health Research from the Consent Provisions of POPIA*, Potchefstroom Electronic L. J., Sept. 2021, 24; Donrich Thaldar, *Why POPIA Does Not Apply to DNA*, S. Afr. J. Sci., Jun. 2021, 117.

18
*Aldine Timber Company v. Hlatwayo* 1932 TPD 337 (S. Afr.); *S v. Riekert* 1977 (3) SA 181 (T) (S. Afr.).

19
*McCarthy Retail Ltd v. Shortdistance Carriers CC* [2001] ZASCA 14 (S. Afr.); *Kudu Granite Operations (Pty) Ltd v. Caterna Ltd* [2003] ZASCA 64 (S. Afr.).

20 Hannah Gaskill, *Judge Weighing Motion to Dismiss Henrietta Lacks’ Family Lawsuit Against Biotech Firm,*Maryland Matters (May 17, 2022) https://www.marylandmatters.org/2022/05/17/judge-weighing-motion-to-dismiss-henrietta-lacks-family-lawsuit-against-biotech-firm/ (Accessed on 3 April 2023).

21
*K v. Minister of Safety and Security* 2005 (6) SA 419 (CC) (S. Afr.).

